# The Feasibility of Ibrexafungerp for the Treatment of Fungal Infections in Patients with Hematological Malignancies

**DOI:** 10.3390/jof8050440

**Published:** 2022-04-23

**Authors:** Justinas Daraskevicius, Vidmantas Petraitis, Linas Davainis, Andrius Zucenka

**Affiliations:** 1 Faculty of Medicine, Institute of Clinical Medicine, Vilnius University, 03101 Vilnius, Lithuania; linas.davainis@santa.lt (L.D.); andrius.zucenka@santa.lt (A.Z.); 2 Department of Medicine, Division of Infectious Diseases, Transplantation-Oncology Infectious Diseases Program, Weill Cornell Medicine of Cornell University, New York, NY 10065, USA; vip2007@med.cornell.edu

**Keywords:** ibrexafungerp, fungal infections, hematological malignancies

## Abstract

Invasive fungal diseases (IFD) remain a major cause of morbidity and mortality in hematological patients, especially those undergoing hematopoietic stem cell transplantation (HSCT). Despite relatively high incidence, diagnosis and treatment remain a challenge due to non-specific manifestation and limited antifungal armamentarium. A first-in-class triterpenoid antifungal ibrexafungerp that acts by inhibiting the glucan synthase enzyme in the fungal cell wall was recently approved by the USA Food and Drug Administration for the treatment of vaginal yeast infections. Preclinical data show activity against numerous fungi species, including azole- and echinocandin-resistant strains. Preliminary data from ongoing phase 3 studies in IFD have been encouraging, but the role of ibrexafungerp in hematological patients who develop fungal infections has not yet been described. Herein, we discuss the feasibility of oral ibrexafungerp-based antifungal therapy for adult patients with hematological malignancies who have either undergone HSCT or received treatment with a novel targeted therapy agent. We present four clinical cases where ibrexafungerp alone or in combination with other antifungal agents was successfully employed for the management of refractory fungal infection. We describe real-life experiences showing the potential clinical implementation of ibrexafungerp for patients with hematological malignancies for the first time, and provoke future discussion.

## 1. Introduction

Invasive fungal diseases (IFD) remain a major cause of morbidity and mortality in hematological patients, especially in those undergoing hematopoietic stem cell transplantation (HSCT) [1,2]. Recipients of HSCT are at the highest risk of developing fungaemia of all cancer patients in Europe [3]. The 12-month cumulative incidence of IFD has reached 8 to 10% after allogeneic HSCT, with rates as high as 17% in patients undergoing haploidentical HSCT [4,5]. Immunosuppression with profound and prolonged neutropenia, along with acute graft-versus-host disease (GVHD) are the major factors leading to the increases in susceptibility to fungal infections in this population [6]. Despite its relatively high incidence, the diagnosis and treatment of IFD remain a challenge due to its non-specific manifestation and limited antifungal armamentarium. Currently, the azole class is the only orally available antifungal therapy; however, this has several limitations, including drug–drug interactions with novel targeted agents. Furthermore, fungal resistance is emerging globally, and oral broad-spectrum antifungals that are safe and well tolerated are greatly needed for hematological patients who develop fungal infections.

The novel oral antifungal drug of ibrexafungerp, a first-in-class triterpenoid antifungal that acts by inhibiting the glucan synthase enzyme in the fungal cell wall, was recently approved by the USA Food and Drug Administration for the treatment of vaginal yeast infection. Preclinical studies have shown ibrexafungerp’s high efficacy against numerous fungal species, including echinocandin- and azole-resistant strains [7]. Several clinical trials are ongoing to evaluate ibrexafungerp’s efficacy and safety in the setting of invasive *Candida* and *Aspergillus* infections. The preliminary data from ongoing phase 3 studies in invasive fungal infections have been encouraging [8].

Herein, we discuss the feasibility of oral ibrexafungerp-based antifungal therapy for adult patients with hematological malignancies who have either undergone HSCT or received novel targeted therapy agents. We present our single-center experience of four different clinical cases where ibrexafungerp alone or in combination with other antifungals was successfully employed in the management of fungal infection. All patients were treated at Vilnius University Hospital Santaros Klinikos, and gave informed consent for treatment and data collection.

### 1.1. CASE 1

A 72-year-old unfit male was diagnosed with secondary acute myeloid leukemia (AML) and was unsuccessfully treated with low-dose cytarabine (LDAraC) in combination with glasdegib. Salvage therapy with venetoclax, LDAraC, and actinomycin D (ACTIVE) induced complete remission (CR). However, during the treatment, asymptomatic funguria and elevated C-reactive protein (CRP) values were observed with a *Pichia kudriavzevii* (synonymously known as *Candida krusei*) identification in the patient’s urine culture. The patient was neutropenic (ANC 300 cells/mm^3^), so inpatient treatment with IV micafungin 100 mg QD was started. On the seventh day of treatment, the urine culture was negative and the CRP values were steadily decreasing, so IV micafungin was discontinued and the patient was discharged. However, 3 weeks later, a recurrence of *Candida krusei* in the urine was detected despite the absence of common risk factors such as a bladder catheter. Due to poor general health, impaired kidney function, and the patient’s will to continue outpatient therapy, oral ibrexafungerp 750 mg QD was started as an alternative to azoles to avoid any potential interactions with venetoclax. Declining CRP values and negative urine culture were noted on the ninth day of ibrexafungerp monotherapy. After a second negative urine culture (on the 16th day of treatment) ibrexafungerp was discontinued. The patient remains in CR receiving anti-leukemic maintenance therapy with no further fungi isolation from microbiological samples.

### 1.2. CASE 2

High-risk myelodysplastic syndrome (HR-MDS) was diagnosed for a 70-year-old male patient. Due to disease progression after decitabine therapy, the salvage ACTIVE regimen was administered followed by HSCT from a matched sibling. However, a relapse was confirmed 5 months post-HSCT, and ACTIVE treatment was re-initiated. Despite antifungal prophylaxis with IV micafungin 150 mg QOD, the patient developed sepsis caused by *Candida krusei* on the 12th day of salvage therapy. Based on antifungal susceptibility testing using the concentration gradient Etest strip technique, IV liposomal amphotericin B 200 mg QD was added to IV micafungin 150 mg QD, and administered for 18 days. However, a fever up to 38 °C and an increase in CRP were observed during treatment; thus, monotherapy with oral ibrexafungerp 750 mg QD was initiated. The fever resolved and no were fungi isolated from the blood or stool (negative culture on the fifth day of ibrexafungerp). CRP levels remained elevated during follow-up, although this was attributed to HR-MDS progression. The patient did not develop any further fungal infection, but unfortunately died of HR-MDS progression after several months.

### 1.3. CASE 3

AML with a monoallelic mutation of *CEPBA* was diagnosed in a 60-year-old male who was successfully treated with 7 + 3 induction and allogeneic HSCT from a matched (HLA 10/10) unrelated donor. After 9 months, a relapse occurred and salvage therapy with ACTIVE was initiated. After the first cycle, CR with incomplete platelet recovery was achieved, and the patient proceeded to a second allogeneic HSCT. On day +73 post-HSCT, the patient complained about joint (right knee and right elbow) pain and swelling. Microbiological examination of the synovial fluid confirmed fungal arthritis caused by *Candida krusei*. No clinical improvement was evident after the initial IV anidulafungin 100 mg QD therapy, and increasing CRP levels were noted. The treatment was changed to IV micafungin 200 mg QD concomitantly with IV liposomal amphotericin B 200 mg QD. After 16 days of dual antifungal therapy, the clinical effect was subtle: CRP remained high, as did the fever, and *Candida krusei* persisted in the synovial fluid culture. Based on poor general health and a lack of clinical improvement, oral ibrexafungerp 750 mg QD was started concomitantly with liposomal amphotericin B 200 mg QD. The joint pain and fever resolved during the first 5 days of treatment, and the CRP levels decreased significantly (113 → 64 → 34 mg/L). Due to the clinical improvement, control arthrocentesis was not performed. After 15 days of combination treatment, the liposomal amphotericin B was discontinued to minimize drug-related toxicity, and ibrexafungerp 750 mg QD was continued with no recurring fungal infections. The patient died due to refractory graft-versus-host disease after several months.

### 1.4. CASE 4

A 65-year-old male was diagnosed with *FLT3*-mutated AML and underwent frontline 7 + 3 induction and salvage ACTIVE + gilteritinib therapy before proceeding to matched unrelated donor allogeneic HSCT. Four months post-HSCT, during the maintenance therapy with gilteritinib, the patient developed dyspnea, cough, and a fever with high CRP values. Invasive pulmonary aspergillosis was confirmed based on a chest CT, positive BAL galactomannan (0.63 index, positive >0.5), and the biopsy results, though multiple BAL cultures were negative. Despite the front-line therapy with micafungin 150 mg QD, second-line isavuconazole 200 mg QD, and third-line liposomal amphotericin B 150 mg QD in combination with inhaled voriconazole 40 mg TID, only minimal improvement was evident. Thus, oral ibrexafungerp 750 mg QD was added to liposomal amphotericin B and inhaled with voriconazole for a total of 41 days. Triple antifungal therapy resulted in the resolution of the fever and dyspnea, and improved the cough. Two negative BAL galactomannan results, the partial decline in CRP, and the absence of hyphae histologically allowed the patient to be discharged. The patient had complained about worsening nausea during therapy, possibly related to ibrexafungerp, but otherwise the treatment was well tolerated. A lack of radiological improvement and the elevation of CRP were attributable to the residual pulmonary granulomatous inflammation.

## 2. Discussion

The epidemiology of fungal infections in patients with hematological malignancies has changed significantly since the development of azoles. Azole-based antifungal prophylaxis has been widely employed in high-risk neutropenic patients, substantially reducing rates of *Candida* infections. However, despite significantly lower rates of invasive candidiasis (IC), *Candida* species are becoming more and more resistant to azoles, as well as echinocandins [9]. In 2010, the Transplant-Associated Infection Surveillance Network implicated *Candida glabrata* as the most common cause of IC among HSCT recipients in 23 US transplant centers [4]. There is now a slightly more diverse epidemiological landscape, as multidrug-resistant *Candida auris* is emerging globally [10]. Additionally, the use of azole-based prophylaxis has led to an increase in mold infections, with *Aspergillus* spp. becoming a predominant cause of IFD. Invasive aspergillosis (IA) has a dismal prognosis among HSCT recipients, with an 84% mortality rate [11]. The European Conference on Infections in Leukemia (ECIL) has recommended voriconazole or isavuconazole as a first line of treatment for patients diagnosed with IA, but treatment results are poor [12].

The novel triterpenoid of antifungal ibrexafungerp addresses this resistance problem, showing promising activity against multidrug-resistant *Candida* species, such as *C. glabrata* and *C. krusei* [13]. Furthermore, an interim analysis of the phase 3 CARES study has shown encouraging results, with a complete response rate reaching 80% (8/10) in patients with invasive candidiasis or candidemia due to *Candida auris* [14]. *Candida* spp. and *Aspergillus* spp. are both susceptible to ibrexafungerp, based on in vitro studies [15]. Moreover, Petraitis et al. have shown the superiority of ibrexafungerp and isavuconazole combination therapy over monotherapy in experimental invasive pulmonary aspergillosis, providing a rationale for ibrexafungerp combinations with conventional antifungals in clinical settings [16].

Our clinical experience reflects the potential activity of ibrexafungerp in high-risk hematological patients with refractory fungal infections caused by *Candida krusei* or *Aspergillus* spp. In all cases, signs of clinical improvement, negative cultures or biopsies, and declining CRP values were only evident after starting ibrexafungerp. The failure of previous therapy could be attributable to drug-resistant strains or the low tissue penetration of the antifungal treatment. Of note, the occurrence of the uncommon *Cancida krusei* infections may also be related to the previous use of antifungals. Two of our patients received ibrexafungerp monotherapy, whereas ibrexafungerp was administered in combination with liposomal amphotericin B in other cases (Case 3 and Case 4). The latter two cases support the idea that the possible synergy between ibrexafungerp and polyenes could overcome the fungal resistance problem and the improve outcomes of patients who develop IFD, including those with invasive aspergillosis.

Currently, echinocandins are recommended for front-line therapy against IC in neutropenic patients due to their excellent activity against *Candida* spp. [12]. Despite moderate hepatotoxicity, echinocandins are otherwise well tolerated and can be safely employed in hematological patients. However, echinocandins can only be administered intravenously, creating challenges for treatment in the outpatient setting. Our first presented patient (Case 1) had general poor health; thus, our goal was to minimize treatment-related toxicities and avoid unnecessary hospitalization. We started outpatient ibrexafungerp, which was well tolerated and effective, with no fungi isolation on the ninth day of treatment. This case illustrates one of the major advantages of ibrexafungerp: the broad-spectrum applicability of its oral formulation.

Drug–drug interactions should also be considered. The use of conventional antifungals in hematological patients is complicated, as a number of drugs are metabolized via *CYP3A*. The azole antifungal inhibitory effect on the *CYP450* family of isoenzymes requires dose adjustment and monitoring when co-administered with *CYP* substrates. Although ibrexafungerp is a substrate of *CYP3A4* and a potential inhibitor of *CYP2C8*, it overcomes major *CYP*-mediated drug interactions in vivo, resulting in the safer and easier coadministration of ibrexafungerp and other *CYP3A4* substrates frequently used in HSCT, such as tacrolimus or cyclosporin A [17]. Two of our patients developed fungal infections during anti-leukemic treatment with venetoclax, a *CYP3A4* substrate; thus, azoles were not administered to avoid venetoclax dose reductions due to potential drug–drug interactions. Both of our presented patients received ibrexafungerp concomitantly with standard doses of venetoclax, and neither adverse events nor toxicities were observed, providing support for ibrexafungerp’s safety and efficacy when used concomitantly with novel agents. This fact is particularly important, as novel treatment strategies based on targeted therapies are emerging.

Our real-life experience shows the potential clinical implementation of ibrexafungerp in patients with hematological malignancies, and provokes future discussion. We represent four clinical cases where ibrexafungerp, either as a monotherapy or in combination with liposomal amphotericin B, was effective for the treatment of fungal infections when alternative antifungal therapies had previously failed, or had to be avoided due to possible treatment-related toxicities or drug–drug interactions. However, we did not aim to evaluate the effectiveness or safety of ibrexafungerp, and we acknowledge that our findings are insufficient for general conclusions. For the first time, we suggest that ibrexafungerp may be considered as an add-on or monotherapy for the treatment of fungal infections in hematological patients, especially for those undergoing HSCT. Thus, this warrants further clinical trials.

## Data Availability

Not applicable.
